# Dietary *Bacillus licheniformis* shapes the foregut microbiota, improving nutrient digestibility and intestinal health in broiler chickens

**DOI:** 10.3389/fmicb.2023.1113072

**Published:** 2023-02-10

**Authors:** Yunsheng Han, Xin Xu, Jiaxin Wang, Hongying Cai, Daojie Li, Hongwei Zhang, Peilong Yang, Kun Meng

**Affiliations:** ^1^Key Laboratory of Feed Biotechnology of Ministry of Agriculture and Rural Affairs, Institute of Feed Research, Chinese Academy of Agricultural Sciences (CAAS), Beijing, China; ^2^National Engineering Research Center of Biological Feed, Beijin, China; ^3^Chengde Academy of Agricultural and Forestry Sciences, Chengde, China

**Keywords:** *Bacillus licheniformis* BCG, broiler, digestive enzyme activity, nutrient transporter, ileac and cecum microbiota, intestinal inflammation, tight junction

## Abstract

*Bacillus licheniformis* is considered a potential alternative to antibiotic growth promoters of animal growth and health. However, the effects of *Bacillus licheniformis* on the foregut and hindgut microbiota, and their relationships with nutrient digestion and health, in broiler chickens remain unclear. In this study, we aimed to identify the effects of *Bacillus licheniformis* BCG on intestinal digestion and absorption, tight junctions, inflammation, and the fore- and hind-gut microbiota. We randomly assigned 240 1-day-old male AA broilers into three treatment groups: CT (basal diet), BCG1 (basal diet + 1.0 × 10^8^ CFU/kg *B. licheniformis* BCG), and BCG2 (basal diet + 1.0 × 10^9^ CFU/kg *B. licheniformis* BCG). On day 42, the jejunal and ileal chyme and mucosa were subjected to analysis of digestive enzyme activity, nutrient transporters, tight junctions, and signaling molecules associated with inflammation. The ileal and cecal chyme were subjected to microbiota analysis. Compared with the CT group, the *B. licheniformis* BCG group showed significantly greater jejunal and ileal α-amylase, maltase, and sucrase activity; moreover, the α-amylase activity in the BCG2 group was higher than that in the BCG1 group (*P* < 0.05). The transcript abundance of FABP-1 and FATP-1 in the BCG2 group was significantly greater than that in the CT and BCG1 groups, and the GLUT-2 and LAT-1 relative mRNA levels were greater in the BCG2 group than the CT group (*P* < 0.05). Dietary *B. licheniformis* BCG resulted in significantly higher ileal occludin, and lower IL-8 and TLR-4 mRNA levels than observed in the CT group (*P* < 0.05). *B. licheniformis* BCG supplementation significantly decreased bacterial community richness and diversity in the ileum (*P* < 0.05). Dietary *B. licheniformis* BCG shaped the ileac microbiota by increasing the prevalence of *f_Sphingomonadaceae, Sphingomonas*, and *Limosilactobacillus*, and contributed to nutrient digestion and absorption; moreover, it enhanced the intestinal barrier by increasing the prevalence of *f_Lactobacillaceae, Lactobacillus*, and *Limosilactobacillus*. Dietary *B. licheniformis* BCG decreased microbial community diversity by diminishing *Desulfovibrio, Alistipes, Campylobacter, Vibrio, Streptococcus*, and *Escherichia coli*-Shigella levels, and down-regulating inflammatory associated molecule expression. Therefore, dietary *B. licheniformis* BCG contributed to digestion and absorption of nutrients, enhanced the intestinal physical barrier, and decreased intestinal inflammation in broilers by decreasing microbial diversity and optimizing the microbiota structure.

## 1. Introduction

In poultry production, broiler chickens are generally subjected to harsh and stressful conditions, particularly when they are reared at high stocking density, thus resulting in host stress and immune dysfunction (Kridtayopas et al., 2019). This subhealth status decreases the growth potential and causes intestinal dysfunction in broilers, and increases the economic costs of rearing. The intestines not only digest and absorb nutrients, but also are the largest immune organ (Zhang et al., 2022a). Trillions of microorganisms colonize the gastrointestinal tract; the total microbial number is 10 times the number of host somatic cells, and the collective number of genes is 150 times that in the host genome (Collins et al., 2012; Strandwitz, 2018). The intestinal microbiota co-develops with the host and participates in nutrient digestion, improves intestinal development and health, and regulates the body’s metabolism and immunity function (Li et al., 2019; Han et al., 2020). Gut microbiota disorders have been associated with subhealth and disease. Broilers show a shift in the gut microbial profile when they experience subclinical forms of necrotic enteritis: the relative abundance of *Firmicutes, Lactobacillus*, and *Bacteroides* decreases, thus resulting in low host productivity (Antonissen et al., 2016). Other research has indicated that challenge with *Salmonella typhimurium* decreased *Lactobacillus* prevalence, damaged intestinal morphology, and subsequently decreased the growth performance of broilers (Jazi et al., 2019). The overall consensus is that the intestinal microbiota is interlinked with intestinal health and poultry growth.

Probiotics have been demonstrated to be an effective means of promoting animal growth and improving body health in the post-antibiotic era (Mingmongkolchai and Panbangred, 2018). *Bacillus licheniformis* (*B. licheniformis*) is a Gram-positive bacterium characterized by resistance to stresses such as high acidity and temperature. Because of these characteristics, *B. licheniformis* can be used in livestock production practices. *Bacillus* spores are metabolically dormant under harsh conditions including feed pelleting but subsequently grow in the favorable environment of the gastrointestinal tract after ingestion (Konieczka et al., 2018). *B. licheniformis* shows growth-promoting effects in poultry (Gadde et al., 2017). Moreover, *B. licheniformis* benefits broilers by protecting against heat stress and preventing necrotic enteritis (Abdelqader et al., 2020; Xu et al., 2021). These benefits might be attributable to a variety of biologically active substances produced by *B. licheniformis*, which contribute to feed digestibility, immune system regulation, and enhanced intestinal barrier function (Kim et al., 2004; Zhou et al., 2016; Kan et al., 2021). *B. licheniformis* improves the intestinal mechanical barrier and decreases intestinal permeability by up-regulating the gene expression of mucins and tight junction proteins in laying hens (Wang et al., 2017). Diets containing a mixture of *B. licheniformis* and *B. subtilis* have been found to alleviate *Escherichia coli*-induced enteritis by increasing intestinal epithelial barrier integrity (Yang et al., 2016). The potential underlying mechanism involves regulation of the composition of the intestinal microbiota to restore and maintain intestinal homeostasis (Sanders et al., 2019; Zhao et al., 2022b). For instance, probiotic *Bacillus* strains have been found to prevent or diminish gut colonization by *Chlamydia psittaci, Escherichia coli, Streptococcus*, and *Salmonella*, thus improving intestinal mucosa integrity and gut health (Zuo et al., 2020; Haque et al., 2021). Therefore, probiotic *Bacillus* appears to prevent disease or stress, and promote growth performance, possibly through an optimized intestinal microbial structure and improved gut health.

Although *B. licheniformis* has great potential application value in the broiler industry, the efficacy of probiotic *Bacillus* varies among strains and depends on the exogenous environmental conditions to which animals are exposed (Konieczka et al., 2022). In the present study, we hypothesized that dietary *B. licheniformis* BCG might alter the ileal and cecal microbiota, and contribute to broiler digestibility and gut health. To this end, we aimed to explore the protective roles of *B. licheniformis* BCG involving improved nutrient digestion and absorption, a strengthened intestinal barrier, and decreased inflammation, and to understand their relationships with the gut microbiota shifted by *B. licheniformis* BCG. Our data provided a theoretical basis for application of *B. licheniformis* BCG in the production of healthy broilers.

## 2. Materials and methods

The experimental animal protocol for this study was conducted in accordance with the recommendations of “Guidelines on Welfare and Ethical Review for Laboratory Animals” (GB/T 35892-2018), and approved by the Institutional Animal Care and Use Committee of the Institute of Feed Research of Chinese Academy of Agricultural Sciences (FRI-CAAS20210827).

### 2.1. Animals and experimental design

A total of 240 1-day-old male Arbor Acre broilers (body weight, 42.62 ± 0.82 g) were randomly allocated to three groups. Each group consisted of eight replicates (pens) with 10 broilers per pen. Two phase non-medicated basal diets in mashed form were formulated based on the nutrient requirements of the National Research Council (1994); (Table 1). The three groups included basal diet (CT, *n* = 8), and basal diet with a dose of 1.0 × 10^8^ CFU/kg (BCG1, *n* = 8) and 1.0 × 10^9^ CFU/kg (BCG2, *n* = 8) *B. licheniformis* BCG, respectively (Wang et al., 2017; Kan et al., 2021; Xu et al., 2021). All broilers were feed in wire-floored cages in a one-level battery on their respective diets. The study lasted 42 days, during which time broilers had *ad libitum* to feed and fresh water. Broilers were housed in an environmentally controlled room and temperature was gradually reduced from 35°C on day 1 to 26°C at day 21 and then kept roughly constant. A 20 h light-4 h dark cycle was carried out throughout the experimental period. *B. licheniformis* BCG was isolated from humus soil in the northeast forest area and preserved in the Key Laboratory of Feed Biotechnology of Ministry of Agriculture and Rural Affairs. It presents great biological characteristics in carbohydrate metabolism enzymes and stress tolerance through the whole genome sequencing and *in vitro* evaluation. *B. licheniformis* BCG with viable count = 1.08 × 10^10^ CFU/g was used and mixed in the basal diet, which was prepared in bacterial mashed form after processed in activation, culture, centrifugation, freeze-drying, and grinding.

**TABLE 1:** Ingredients and chemical compositions of experimental diets (as-fed basis).

Ingredient (%)	Content	
	Day 1-21	Day 22-42
Corn	54.88	58.00
Soybean meal	36.27	32.79
Fish meal	1.75	1.33
Soybean oil	3.08	4.28
Dicalcium phosphate	1.24	1.08
Limestone	1.21	1.14
Sodium chloride	0.30	0.30
DL-Methionine	0.15	0.08
L-Lysine-HCl	0.12	0.00
Vitamin and mineral premix^†^	1.00	1.00
**Nutrient composition^‡^**		
Metabolizable energy (MJ/kg)	12.35	12.80
Crude protein (%)	21.86	20.34
Calcium (%)	1.00	0.90
Total Phosphorus (%)	0.71	0.64
Available phosphorus (%)	0.40	0.35
Methionine (%)	0.46	0.37
Methionine + Cysteine (%)	0.75	0.64
Lysine (%)	1.14	0.95

^†^A vitamin-mineral premix provided the following nutrients per kg of diet: vitamin A, 10000 IU; vitamin D, 2000 IU; vitamin E, 20 IU; vitamin K, 1 mg; vitamin B1, 2 mg; riboflavin, 8 mg; vitamin B_12_, 0.01 mg; pantothenic acid, 10 mg; niacin, 35 mg; pyridoxine, 3.5 mg; biotin, 0.2 mg; folic acid, 0.6 mg; Fe, 100 mg; Cu, 10 mg; Mn, 120 mg; Zn, 100 mg; I, 0.7 mg; Se, 0.3 mg.

^‡^Nutrient levels were calculated.

### 2.2. Sample collection

At 42 days of age, after fasting overnight, one broiler representing the average weight from each replicate was selected and humanely slaughtered. Jejunum, ileum and cecum segments were divided and fresh ileal and cecal contents were collected for α-amylase and microbiota analysis. Jejunal and ileal mucosa were scraped by autoclaved blade after precooled saline flush for maltase, sucrase and gene expression analysis. All samples were obtained as described previously (Wang et al., 2008), and immediately frozen in liquid nitrogen and stored at −80°C.

### 2.3. Biochemical analysis

Appropriately 100 mg frozen mucosa and chyme of jejunum and ileum, respectively, were taken and mixed with 1 mL cold buffer (pH7.4), containing 10 mM Tris-HCl, 0.1 EDTA-Na_2_ and 0.8% (w/v) NaCl, and homogenized using an Ultra-Turrax homogenizer for 30 s. Homogenates were centrifuged at 3,000 × *g* for 15 min at 4°C and supernatants transferred to new tubes for protein assay and other measurements. The activities of α-amylase, maltase and sucrase were measured by colorimetry using the commercial kits (Nanjing Jiancheng Bioengineering Institute, Nanjing, China) according to the manufacturer’s protocols. Maltase and sucrase were normalized to tissue protein concentrations, which were measured with a bicinchoninic acid commercial kit (Thermo Fisher Scientific, Waltham, MA, USA) following the manufacturer’s instructions.

### 2.4. Real-time quantitative PCR (RT-qPCR)

Selected mRNA abundance was determined by RT-qPCR, including nutrient transporters genes *FABP-1* (fatty acid binding protein 1), *FATP-1* (fatty acid transport protein 1), *GLUT-2* (glucose transporter 2), *LAT-1* (L type amino acid transporter 1), *PepT-1* (peptide transporter 1) and *SGLT-1* (sodium glucose co-transporter 1), inflammatory molecules’ genes *TLR-4* (Toll-like receptor 4), *IL-1β* (interleukin 1β), *IL-8, IL-10, TNF-α* (tumor necrosis factor α), *TGF-β* (Transforming growth factor β) and *NF-κB* (Nuclear factor kappa B), and tight junction genes *Claudin-1, Occludin, ZO-1* and *Mucin-2*. Total RNA was isolated from ileal mucosa samples (approximately 0.75 mg) using an RNAprep pure tissue kit (Tiangen Biotech Co. Ltd., Beijing, China) under the manufacturer’s instructions. Total RNA concentrations and quality were assessed using a NanoDrop 2000 spectrophotometer (Thermo Fisher Scientific, Waltham, MA, USA). RNA integrity was evaluated using agarose gel (1%) electrophoresis. Then, cDNA was synthesized from 1 μg total RNA using a PrimeScript RT reagent kit (TaKaRa Biotechnology Co., Ltd., Otsu, Japan) following to manufacturer’s protocols. Selected mRNA reactions were detected in 10 μL (Bio-Rad Laboratories, Hercules, CA, USA) using SYBR^®^ Premix Ex TaqTM II (Tli RNaseH Plus) (TaKaRa Biotechnology Co., Otsu, Japan). The primers for nutrient transporters, inflammatory, and tight junction-related and housekeeping genes [glyceraldehyde 3-phosphate dehydrogenase (GAPDH)] were described previously (Wang et al., 2016, 2020). The 2^–ΔΔCt^ method was used for quantification using GAPDH as a reference gene, and relative abundance was normalized to CON group values.

### 2.5. Ileal and cecal microbiota and analysis

Bacterial genomic DNA was extracted from ileal and cecal chyme samples (Qiagen DNA stool mini kit, Qiagen, Germany). DNA quantity and quality were assessed using a NanoDrop 2000 spectrophotometer (Thermo Fisher Scientific, Waltham, MA, USA) and 1% agarose gels, respectively. The V3–V4 hypervariable region 16S rRNA was amplified using specific primers (forward 5′-ACTCCTACGGGAGGCAGCA-3′ and reverse 5′-GGACTACHVGGGTWTCTAAT-3′), containing unique barcodes. Polymerase chain reaction (PCR) was conducted in a total volume of 20 μL, including 1 × FastPfu buffer, 250 μM dNTP, 0.2 μM each primer, 1 U FastPfu polymerase (Beijing TransGen Biotech, Beijng, China), and 10 ng template DNA. PCR products were electrophoresed on 2% agarose gels and purified using a Qiagen gel extraction kit (Qiagen, Germany). Sequencing libraries were constructed using a TruSeq^®^ DNA PCR-Free Sample Preparation Kit (Illumina, San Diego, CA, USA) based on manufacturer’s instructions, and index codes were added. Library quality was assessed using a Qubit V.2.0 Fluorometer (Thermo Fisher Scientific, Waltham, MA, USA). Qualified DNA libraries were loaded into a NovaSeq platform capable of 2 × 250 bp paired-end sequencing reads (Novogene, Beijing, China).

Paired-end reads were generated and merged using FLASH software (V1.2.7)^1^. Operational taxonomic units with 97% identity were gathered using Uparse^2^ (ver. 7.1). Taxonomic annotations were performed using the Mothur algorithm (70% confidence) in the Silva database^3^. Alpha-diversity was analyzed using Observed_species, Chao1, and Shannon indices. Beta-diversity was visualized using principal coordinate analysis (PCoA) plots based on weighted UniFrac distance. Bacterial biomarkers between groups were displayed using the linear discriminant analysis effect size (LEfSe, linear discriminant analysis (LDA) > 3.5).

### 2.6. Statistical analysis

Statistical analyses were performed using one-way analysis of variance in SAS 9.4 (SAS Institute, Inc., Cary, NC, USA). Each broiler served as statistical unit. Differences between treatment means for enzyme activity and gene expression were evaluated using Duncan’s multiple-range tests. Wilcox test was used for alpha-diversity index. LEfSe, *t*-test, and Metastat analyses were used to test for significant differences between microbiota relative abundance. Results were represented as mean with standard error of mean (SEM) in the tables and the mean with standard error (SE) in the figures, while *P* < 0.05 (*) and *P* < 0.01 (^**^) values were considered statistically and extremely significant, respectively. Bar charts were drafted in Graphpad Prism 7.0 software (GraphPad Software Inc., La Jolla, CA, USA).

## 3. Results

### 3.1. Effects of *B. licheniformis* BCG on jejunal and ileal enzyme activity in broilers

The BCG2 group showed significantly greater jejunal and ileal maltase and sucrase activity, and the BCG1 group showed significantly greater ileal sucrase activity, than the CT group (*P* < 0.05, Table 2). No differences in these two parameters were observed between the BCG1 and BCG2 groups (*P* > 0.05). Dietary *B. licheniformis* BCG resulted in significantly greater α-amylase activity than that in the CT group (*P* < 0.05), and this activity was higher in the BCG2 group than the BCG1 group (*P* < 0.05).

**TABLE 2:** Effects of *B. licheniformis* BCG on enzyme activity in the jejunum and ileum in broilers.

Item	Dietary treatments^1^			SEM^2^	*P*-value
	CT	BCG1	BCG2		
**Jejunum**					
Maltase (U/mg prot)	35.60^b^	51.29^a^	49.07^a^	2.584	0.016
Sucrase (U/mg prot)	43.40^b^	53.11^ab^	64.63^a^	2.844	0.003
α-amylase (U/dL)	49.35	52.90	50.26	1.581	0.663
**Ileum**					
Maltase (U/mg prot)	134.50^b^	169.23^a^	178.36^a^	6.953	0.013
Sucrase (U/mg prot)	100.87^b^	129.09^a^	127.31^a^	5.088	0.028
α-amylase (U/dL)	24.61^c^	32.53^b^	44.76^a^	2.220	< 0.01

^a–c^Different superscript letters in a row indicate a significant difference (*P* < 0.05). ^1^CT, control group, basal diet; BCG1, basal diet supplemented with *B. licheniformis* BCG at 1.0 × 10^8^ CFU/kg; BCG2, basal diet supplemented with *B. licheniformis* BCG at 1.0 × 10^9^ CFU/kg. ^2^SEM, standard error of the mean, *n* = 8.

### 3.2. Effects of *B. licheniformis* BCG on nutrient transporter gene mRNA levels in the ileum

**FIGURE 1:**
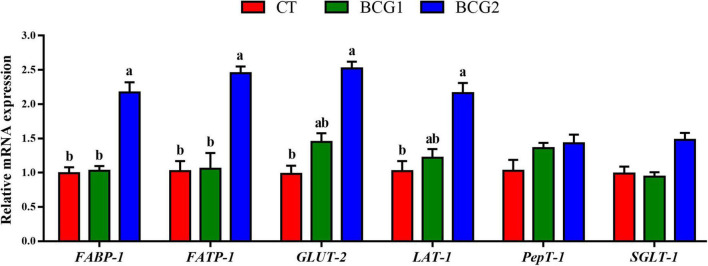
Effects of *Bacillus licheniformis* BCG on nutrient transporter gene mRNA levels in the ileum. Values are mean ± SE (*n* = 6). ^a,b^Bars with different letters within the same index indicate a significant difference between groups (*P* < 0.05).

*FABP-1* and *FATP-1* relative mRNA levels in the BCG2 group were significantly higher than those in the CT and BCG1 groups (*P* < 0.05, Figure 1). The transcript abundance of *GLUT-2* and *LAT-1* was greater in the BCG2 group than the CT group (*P* < 0.05), and no significant difference was found between the BCG1 group and the other groups (*P* > 0.05).

### 3.3. Effects of *B. licheniformis* BCG on tissue morphology and the mRNA expression of tight junction and inflammatory molecules in the ileum

**FIGURE 2:**
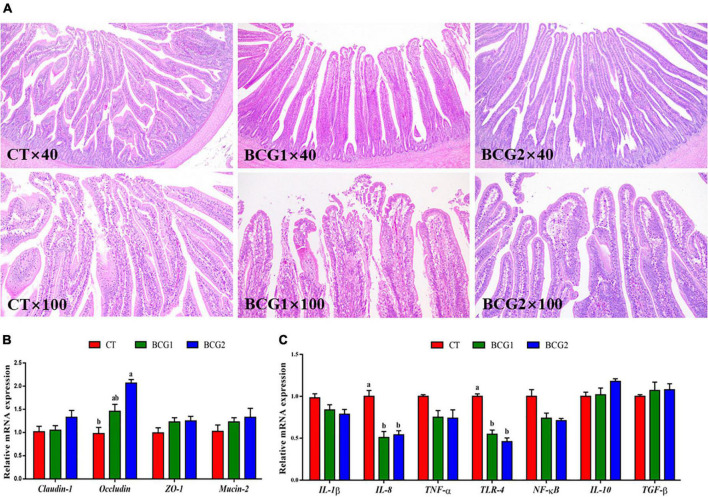
Effects of *Bacillus licheniformis* BCG on ileal morphology, and tight junction and inflammatory molecule gene mRNA levels in the ileum. **(A)** Representative ileal histological sections of broilers. **(B)** mRNA levels of the tight junction genes *claudin-1, occludin, ZO-1*, and *Mucin-2*. **(C)** The mRNA levels of the inflammatory molecule genes *TLR-4, IL-1*β, *IL-8, IL-10, TNF-*α, *TGF-*β, and *NF-*κ*B*. Values are the mean ± SE (*n* = 6). ^a,b^Bars with different letters within the same index indicate a significant difference between groups (*P* < 0.05).

Histological examination of the ileum indicated that the villi and epithelium in the CT group, as compared with the BCG1 and BCG2 groups, showed damage; however, no clear infiltration of inflammatory cells was observed among groups (Figure 2A). Dietary *B. licheniformis* BCG significantly up-regulated ileal *occludin* mRNA levels, which were higher in the BCG2 group than the CT group (*P* < 0.05, Figure 2B). The transcript abundance of *IL-8* and *TLR-4* in the BCG1 and BCG2 groups was lower than that in the CT group (*P* < 0.05, Figure 2C), whereas no significant difference was observed between the BCG1 and BCG2 groups. No significant difference in *claudin-1, ZO-1, mucin-2, IL-1*β, *TNF-*α, *NF*-κ*B, IL-10*, and *TGF-*β transcript abundance was observed between groups (*P* > 0.05).

### 3.4. Effects of *B. licheniformis* BCG on microbiota diversity in the ileum and cecum

**FIGURE 3:**
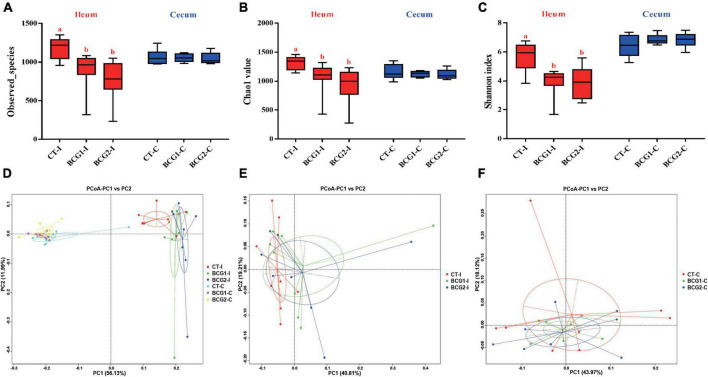
Effects of *Bacillus licheniformis* BCG on alpha- and beta-diversity of the microbiota inhabiting the ileum and cecum in broilers. **(A–C)** Bacterial richness and diversity, estimated with Observed_species, Chao1 value, and Shannon index. **(D–F)** Principal coordinate analysis based on weighted UniFrac distances, showing separation in the microbiota between the ileum and cecum, and between groups in the ileum or cecum. Values are mean ± SE (*n* = 8; BCG1-C, *n* = 7). ^a,b^Boxes with different letters within the same index indicate a significant difference between groups (*P* < 0.05).

A total of 3,917,657 high quality sequencing reads were generated from 47 broiler gut samples, with an average of 69,233 effective sequences/sample. Alpha diversity analyses indicated varying community richness and diversity among groups in the ileal but not the cecal bacterial communities. Both the BCG1 and BCG2 groups showed significantly lower ileal Observed_species, Chao1, and Shannon indexes than the CT group (*P* < 0.05, Figures 3A–C). Differences in the microbial structure among groups and niches were evaluated with PCoA analysis based on weighted UniFrac distance. Microbial communities were well separated between the ileal microbiota and counterparts colonizing the cecum, and between groups in the ileum (Figures 3D–F). ANOSIMs also confirmed the structural dissimilarity between the ileum and cecum, and between the CT group and the two BCG groups in the ileum or cecum (R > 0, *P* < 0.05, Table 3).

**TABLE 3:** ANOSIM analysis of differences in bacterial structure between variables, on the basis of Bray-Curtis distances.

Varibles	*R*-value	*P*-value
CTI VS. CTC	0.8549	0.001
CTI VS. BCG1-C	0.9971	0.001
CTI VS. BCG2-C	0.9967	0.001
CTC VS. BCG1-I	0.7238	0.001
CTC VS. BCG2-I	0.8644	0.002
BCG1-I VS. BCG1-C	0.750	0.001
BCG1-I VS. BCG2-C	0.7812	0.001
BCG2-I VS. BCG1-C	0.9555	0.002
BCG2-I VS. BCG2-C	0.9325	0.001
CTI VS. BCG1-I	0.202	0.029
CTI VS. BCG2-I	0.1663	0.041
BCG1-I VS. BCG2-I	-0.0385	0.669
CTC VS. BCG1-C	0.1764	0.027
CTC VS. BCG2-C	0.1872	0.028
BCG1-C VS. BCG2-C	0.4191	0.001

*R* > 0 represents a significant difference between groups, whereas *R* < 0 indicates a difference within groups greater than that between groups. *P* < 0.05 indicates a significant difference in statistics. CT, control group, basal diet; BCG1, BCG1 group, basal diet supplemented with *B. licheniformis* BCG at 1.0 × 10^8^ CFU/kg; BCG2, BCG2 group, basal diet supplemented with *B. licheniformis* BCG at 1.0 × 10^9^ CFU/kg; I, ileum; C, cecum.

### 3.5. Effects of *B. licheniformis* BCG on ileal and cecal bacterial structures

**FIGURE 4:**
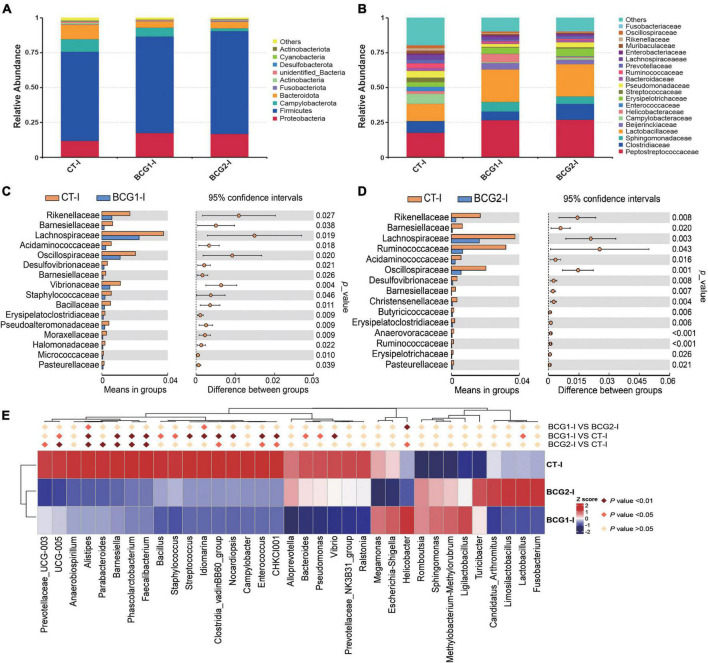
Effects of *Bacillus licheniformis* BCG on bacterial composition in the ileum. **(A,B)** Distribution of ileal bacteria at the phylum and family levels. **(C,D)** The *t*-tests were used to assess significant differences at the family level; *P* < 0.05 indicates a significant difference. **(E)** Statistical analysis of differences in the relative abundance of the top 35 genera; Metastat was used to test for significant differences; light pink diamonds indicate *P* < 0.05, and dark pink diamonds indicate *P* < 0.01 between groups (*n* = 8).

In the ileum, Proteobacteria, Firmicutes, Bacteroidota, and Campylobacterota were the dominant bacterial phyla, with a relative abundance accounting for > 95% of the total ileal bacterial communities (Figure 4A). In contrast to the CT-ileal (CT-I) group, the BCG1-I and BCG2-I groups showed an increase in the relative abundance of Proteobacteria and Firmicutes, from 11.79 to 17.44 (16.81%), and from 64.11 to 69.37 (73.94%), respectively (*P* > 0.05). However, the BCG1-I and BCG2-I groups showed a decrease in the relative abundance of Bacteroidota and Campylobacterota, from 10.25 to 4.19 (4.62%), and from 9.08 to 6.42 (1.98%), respectively (*P* > 0.05). At the family level, in contrast to the CT group, the BCG1 and BCG2 groups showed an increase in the relative abundance of *Peptostreptococcaceae, Sphingomonadaceae*, and *Lactobacillaceae*, from 17.6 to 26.67 (26.92%), from 0.04 to 6.89 (5.39%), and from 12.18 to 23.17 (22.97%), respectively (*P* > 0.05). The relative abundance of *Campylobacteraceae* in the BCG1-I and BCG2-I groups was, respectively, 0.68 and 1.44%, and lower than the 7.31% in the CT-I group (*P* > 0.05, Figure 4B). Diets with BCG1 and BCG2 significantly decreased several low abundance bacteria at the family level (*P* < 0.05, Figures 4C, D). For the 35 most dominant ileal genera, the BCG1 and BCG2 diet groups showed significantly lower relative abundance of *Clostridiales bacterium CHKCI001, Enterococcus, Clostridia_vadinBB60_group, Faecalibacterium, Phascolarctobacterium, Barnesiella, Alistipes*, and *Ruminococcaceae UCG-005* than that in the CT group (*P* < 0.05, Figure 4E). The BCG1 diet group showed significantly lower relative abundance of *Lactobacillus, Vibrio, Pseudomonas, Bacteroides, Streptococcus, Staphylococcus*, and *Bacillus* than that in the CT group (*P* < 0.05). The relative abundance of *Helicobacter* in the BCG2-I group was significantly lower than that in the CT-I and BCG1-I groups (*P* < 0.05). In contrast to the CT-I group, the BCG1-I and BCG2-I groups showed an increase in the relative abundance of *Sphingomonas* from 0.03 to 6.34 (11.55%), whereas *Campylobacter* levels showed a decrease from 7.31 to 0.68 (1.44%) (*P* > 0.05).

**FIGURE 5:**
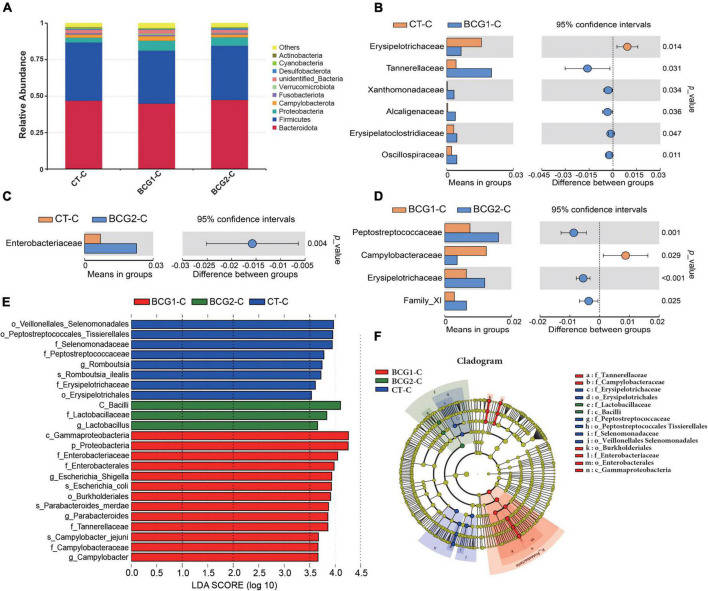
Effects of *Bacillus licheniformis* BCG on bacterial composition in the cecum. **(A)** Distribution of cecal bacteria at the phylum level. **(B–D)** The *t*-tests were used to assess significant differences at the family level; *P* < 0.05 indicates a significant difference. **(E)** LEfSe analysis of differences in taxa enrichment in microbial communities between groups; bacterial taxa with a logarithmic LDA score > 3.5 were biomarker taxa. **(F)** Cladogram showing bacteria with significant differences between groups (*n* = 8; BCG1-C, *n* = 7).

In the cecum, Bacteroidota, Firmicutes, Proteobacteria, Campylobacterota, and Fusobacteriota were the major bacterial phyla, and their relative abundance was seldom affected by BCG treatments (*P* > 0.05, Figure 5A). On the basis of T-test and LEfSe results, CT-C broilers had a higher relative abundance of *f_Erysipelotrichaceae, f_Peptostreptococcaceae*, and *g_Romboutsia* than that in the BCG1-C and BCG2-C groups, and *f_Erysipelotrichaceae* and *f_Peptostreptococcaceae* levels in the BCG2-C group were significantly higher than those in the BCG1-C group (*P* < 0.05, Figures 5B–F). *f-Lactobacillaceae* and *g_Lactobacillus* were the dominant bacteria in the BCG2-C group, in contrast to the BCG1-C and CT-C groups, whereas *f_Enterococcaceae, f_Campylobacteraceae, f_Tannerellacea*e, *g_Escherichia-Shigella, g_Campylobacter*, and *g_Parabacteroides* were the dominant bacteria in the BCG1-C group, in contrast to the CT-C and BCG2-C groups. The *f_Campylobacteraceae* level in the BCG2-C group was significantly lower than that in the BCG1-C group (*P* < 0.05).

### 3.6. Correlations between ileal microbiota and enzyme activity and nutrient transporters or inflammatory and barrier parameters

**FIGURE 6:**
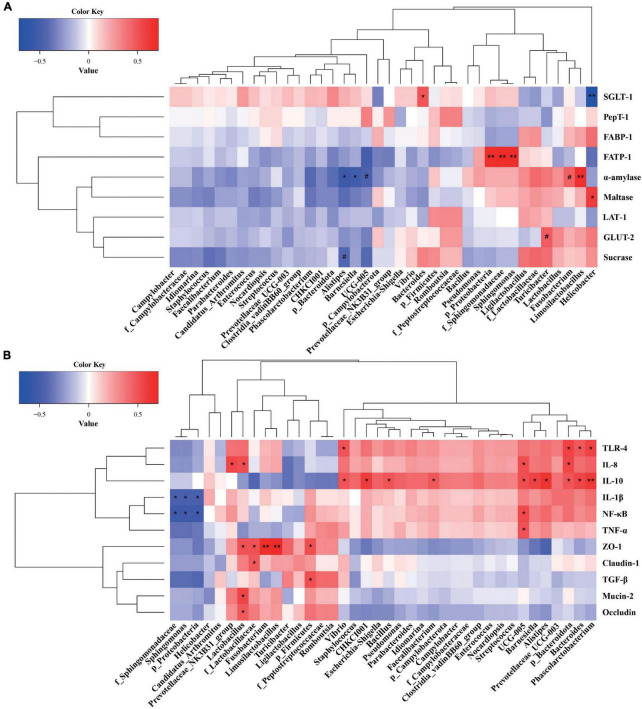
**(A)** Correlations between the ileal microbiota and enzyme activity and nutrient transporters. **(B)** Correlations between the ileal microbiota and inflammatory and barrier parameters. Red represents a positive correlation, and blue represents a negative correlation. *P < 0.05 and **P < 0.01 indicate significant and extremely significant correlations.

A Spearman’s correlation analysis was performed to explore the relationships of predominant ileal phyla, families, and genera with the nutrient digestion and absorption, or inflammatory and barrier parameters (Figure 6). *Proteobacteria, f_Sphingomonadaceae*, and *Sphingomonas* were significantly positively correlated with *FATP-1* expression, whereas *Alistipes* and *Barnesiella* were significantly negatively correlated with α-amylase activity (*P* < 0.05, Figure 6A). *Bacteroides* and *Limosilactobacillus* showed a significant positive correlation with *SGLT-1* expression and α-amylase activity (*P* < 0.05). *Helicobacter* was significantly positively correlated with maltase activity but negatively correlated with *SGLT-1* expression (*P* < 0.05). *Firmicutes, f_Lactobacillaceae, Lactobacillus, Fusobacterium*, and *Limosilactobacillus* showed significant positive correlations with *ZO-1* expression. *f_Lactobacillaceae* and *Lactobacillus* were significantly positively correlated with *occludin* and *mucin-2* expression. *f_Lactobacillaceae* showed a significantly positive correlation with *claudin-1* expression (*P* < 0.05, Figure 6B). *Bacteroidota, Bacteroides*, and *Phascolarctobacterium* were significantly correlated with *TLR 4* and *IL-10* expression, and *Ruminococcaceae UCG-005* was significantly correlated with *TNF-*α, *NF-*κ*B*, and *IL-8* expression (*P* < 0.05). *Proteobacteria, f_Sphingomonadaceae*, and *Sphingomonas* showed significant negative correlations with *IL-1*β and *NF-*κ*B* expression (*P* < 0.05).

## 4. Discussion

*Bacillus* spp. bacteria serve as a potential alternative to antibiotic growth promoters in livestock production, owing to their stress resistance and probiotic characteristics. Previous studies have shown that dietary supplementation with *B. licheniformis* significantly promoted broiler growth by increasing body weight and decreasing the feed to weight ratio (Liu et al., 2012; Chen and Yu, 2020; Xu et al., 2021). In addition, several studies have indicated that *B. licheniformis* administration promoted broiler growth under heat stress and *Clostridium perfringens* challenge conditions (Song et al., 2014; Zhou et al., 2016; Musa et al., 2019). These findings were consistent with those in our previous study indicating that diets with *B. licheniformis* at a dose of 1.0 × 10^9^ CFU/kg significantly increases broiler body weight and average daily gain (Wang et al., 2022). Thus, *B. licheniformis* improved broiler growth performance under both normal and stress conditions. The mechanism underlying the improvements in nutrient digestion and host health were likely to involve the formation of beneficial metabolites such as extracellular digestive enzymes, organic acids, and antibacterial peptides (Kim et al., 2004; Knap et al., 2010; Xu et al., 2021). Thus, the activity of α-amylase, maltase, and sucrase in the jejunum and ileum was further determined, because these enzymes participated in the digestion of nutrients.

In poultry, feed starch is generally degraded by α-amylase into smaller molecular oligomers after initial hydrolyzation by the microbiota, and is finally hydrolyzed into maltose and maltotriose in the small intestine (Dhital et al., 2017). Subsequently, maltose and maltotriose are hydrolyzed into glucose by sucrase, and maltase resides at the surfaces of the intestinal villi (Zhou et al., 2021). *B. licheniformis* secretes extracellular enzymes such as α-amylase and proteases (Kaewtapee et al., 2017). In the present study, dietary *B. licheniformis* BCG, compared with the CT diet, significantly increased jejunal and ileal α-amylase, maltase, and sucrase activity, in partial agreement with findings reported by Yang et al. (2021). The α-amylase activity is an important rate-determining factor in starch digestion, because endogenous α-amylase activity is low in broilers (Zhou et al., 2021). The increased α-amylase activity may increase the nutrient digestibility coefficients of starch and organic matter and consequently increase feed metabolizable energy, thus contributing to broiler growth performance (Jiang et al., 2008; Kaczmarek et al., 2014). In addition, nutrient transporters are crucial for nutrient absorption at the brush border membrane in the small intestine. Both FABP-1 and FATP-1 are important for intestinal absorption of lipids and fatty acids, particularly in long-chain fatty acid metabolism and intracellular transportation (Coe et al., 1999; Richieri et al., 1999). GLUT-2 is an Na^+^-independent transporter responsible for the basolateral exit of glucose from the intestinal mucosa into the portal circulation (Wang et al., 2020). Intestinal LAT-1 participates in branched-chain and aromatic amino acid transport in an Na^+^-independent manner (Broer, 2008). As previously reported, probiotic supplementation enhances expression of some types of nutrient transporters in the small intestines in animals (Faseleh Jahromi et al., 2016; Duan et al., 2018). In agreement with previous findings, the present study indicated that *B. licheniformis* BCG administration significantly increased *FABP-1, FATP-1, GLUT-2*, and *LAT-1* expression, to a greater extent in the BCG2 group than the BCG1 group. Thus, dietary supplementation with *B. licheniformis* BCG may aid in starch digestion and the absorption of glucose, amino acids, and fatty acids, thereby resulting in higher growth performance of broilers.

To determine whether *B. licheniformis* BCG contributed to intestinal health, we assessed the gene expression of TLR4 signaling pathway related molecules, tight junction proteins, and mucin-2. TLR4, as a pathogen-associated molecular pattern, mediates downstream inflammatory signals through the linker protein MyD88, thereby activating NF-kB, which then translocates into the nucleus and elicits pro-inflammatory cytokine secretion and cellular responses of immune-associated cells (Huebener and Schwabe, 2013; Kan et al., 2021). *B. licheniformis* has been demonstrated to decrease secretion of the pro-inflammatory cytokines IL-8 and IL-6 *in vivo* (Deng et al., 2012; Roselli et al., 2017). Pretreatment with *B. licheniformis* has been found to decrease the serum TNF-α and IL-1β levels in an acetaminophen-induced acute liver injury rat model (Neag et al., 2020). Similarly, *B. licheniformis* also decreases the inflammatory response in an LPS-induced acute inflammation rat model (Deng et al., 2017). Moreover, *B. licheniformis* treatment markedly counteracts the increase in *IL-6, IL-8* and *TNF-α* inflammatory gene expression induced by enterotoxigenic *Escherichia coli* F4 *in vitro*. The immunity homeostasis of HT-29 cells is improved by treatment with *B. licheniformis* MCC 2514, on the basis of downregulation of IL-1α, IL-6, IL-8, IL-12, and TNF-α, and upregulation of IL-4, IL-10, TGF-2, and TGF-3 (Rohith and Halami, 2021a). These results are consistent with our observation that the ileal *TLR 4* and *IL-8* expression in the BCG1 and BCG2 groups was significantly lower than that in the CT group. The underlying mechanism was associated with *B. licheniformis* bacteriostasis. As previously reported, *B. licheniformis* inhibited the growth of pathogenic bacteria such as *Kocuria rhizophila* and enterotoxigenic *Escherichia coli* by adhering to the intestines (Rohith and Halami, 2021b; Li et al., 2022a). Another key mechanism through which *B. licheniformis* attenuates inflammation might involve intestinal barrier improvement.

The integrated intestinal barrier plays an important role in maintaining epithelial cell function, because the epithelium is permeable to feed-associated antigens, and luminal pathogens and their toxins (Awad et al., 2017). The transmembrane proteins claudins, occludins, and zonula occludens involved in tight junctions are responsible for connecting epithelial cells and regulating paracellular and intracellular permeability (Aijaz et al., 2006). The regular permeability and integrity of tight junctions are generally negatively affected by stress factors, such as heat stress and pathogen invasion (Song et al., 2014; Musa et al., 2019; Li et al., 2022a). Under *Clostridium perfringens* challenge, dietary *B. licheniformis* significantly increases the gene expression of *claudin-1* and *ZO-1* in the duodenum in broilers at post challenge days 7 and 21 (Musa et al., 2019). In laying hens, *B. licheniformis* administration enhances the intestinal mechanical barrier by upregulating the gene expression of mucin-2 and tight junction proteins (Wang et al., 2017). In *in vitro* experiments, *B. licheniformis* PF9 application has been found to reverse the decrease in ZO-1 and occludin expression in the cell membrane after challenge with enterotoxigenic *Escherichia coli* (Li et al., 2022a). In agreement with previous studies, the present study indicated that a diet with a high dose of *B. licheniformis* BCG resulted in significantly higher *occludin* expression than that in the CT group. Thus, *B. licheniformis* BCG alleviates ileal inflammation partly by enhancing the physical barrier. The improved barrier function resulting from *B. licheniformis* BCG might be attributable to its biologically active substances including bacteriocin and antibacterial peptides, which suppress pathogenic bacterial colonization and balance the intestinal microbiota (Zhou et al., 2016; Kan et al., 2021).

The gut microbiota provides a broad range of functions for hosts, for example, the digestion of complex dietary nutrients, defense against pathogens, enhancement of the intestinal barrier, and promotion of immune maturation (Koh et al., 2016). Owing to differences in histology and function, the broiler intestinal tract is generally divided into the fore- and hindgut. Previous studies in broilers have focused primarily on the hindgut microbiota and their functions, whereas studies on the microbiota in the foregut and their interactions with the host have been limited (Oakley et al., 2014; Huang et al., 2018). In the current study, we analyzed ileal and cecal microbial diversity and composition, and their effects under *B. licheniformis* BCG treatment. The PCoA revealed a clear separation between the ileum and cecum compartments, thus indicating a large difference between them, in agreement with findings from a previous study (van der Wielen et al., 2002). In addition, piglets fed *Bacillus* species probiotics show diminished microbial richness in feces (Kaewtapee et al., 2017; Poulsen et al., 2018). These findings are partially consistent with those in the current study indicating diminished bacterial richness and diversity in the ileum but not cecum in broilers in response to *B. licheniformis* BCG. Our findings indicated that dietary supplementation with *B. licheniformis* BCG shifted the ileal bacterial community structure in broilers. Theoretically, gut bacterial diversification is a gradual process that increases with age, and high diversity is considered a sign of gut bacterial maturity (Micah et al., 2007). Premature formation of an adult-type bacterial community negatively affects host gut immunity (Nylund et al., 2013). Dietary supplementation with *B. licheniformis* BCG significantly decreased ileal inflammation in the present study, a result partially attributed to the decrease in bacterial diversity.

Bacterial composition is also closely associated with host physiology, including digestion, metabolism, and immunity. In the present study, considerable variations in Proteobacteria, Firmicutes, Campylobacterota, and Bacteroidota were found between the ileum and cecum. Oxygen-sensitive Bacteroidota markedly increased from the ileum to the cecum, because it is adapted to a low oxygen environment. However, oxygen-tolerant Proteobacteria and Campylobacterota, such as *Enterococcaceae* and *Campylobacteraceae*, decreased from the ileum to the cecum. Our results are consistent with previous observations that spatial shifts in bacterial composition depend on microenvironment change (Huang et al., 2018; Li et al., 2022b). Firmicutes include *Peptostreptococcaceae, Clostridiaceae*, and *Lactobacillaceae*, which are positively associated with energy intake and have been found to provide an additional 628 kJ of energy when their abundance increases by 20% (Hildebrandt et al., 2009; Jumpertz et al., 2011). This causal relationship has also been found in piglet models (Mach et al., 2015; Li et al., 2022a). Firmicutes fermentation is a more suitable energy source than Bacteroidota because it produces more short chain fatty acids, thus enhancing efficient heat absorption (Krajmalnik-Brown et al., 2012; Pan et al., 2022). We observed that a diet with *B. licheniformis* increased ileal Firmicutes levels but decreased Bacteroidota levels, a finding partially explained by our previous results indicating that *B. licheniformis* administration promoted broiler growth (Wang et al., 2022). In recent studies, *Sphingomonas* involved in the *f*_*Sphingomonadaceae* has been observed as an abundant bacterium in chicken intestines (Chen et al., 2018; Zhang et al., 2022b). The abundance of *Sphingomonas* is significantly positively correlated with fat catabolism in the liver, serum, and muscle (Li et al., 2020; Zhang et al., 2022b). In addition, *Sphingomonas* participates in lipid metabolism across the entire process of chicken embryonic development (Akinyemi et al., 2020). Diets with *B. licheniformis* resulted in significantly greater ileal *f*_*Sphingomonadaceae* and *Sphingomonas* levels than those in the CT group; these levels were significantly positively correlated with *FATP-1* expression but significantly negatively correlated with *IL-1*β and *NF-*κ*B* expression. These findings suggested that the shifts in the prevalence of *f*_*Sphingomonadaceae* and *Sphingomonas* after *B. licheniformis* BCG treatment contributed to the intestinal absorption and oxidative decomposition of lipids and fatty acids, and alleviation of intestinal inflammation.

On the basis of the analysis of the abundance of bacteria in the present study, *Lactobacillus, Bacteroides, Alistipes, Escherichia-Shigella, Desulfovibrio, Streptococcus, Ruminococcaceae_UCG-00*5, *Fusobacterium*, and *Campylobacter* predominated in broilers. In general, the presence of *Lactobacillus* is considered beneficial for intestinal health and animal health, owing to their immunomodulation, pathogen inhibition and bacteriocin production ability (Kaewtapee et al., 2017; Zhao et al., 2022b). In the current study, broilers in the BCG2 group had higher *Lactobacillus* levels in both the ileum and cecum than those in the CT or BCG1 group, in agreement with findings from previous studies (Hung et al., 2019; Zhao et al., 2022b). An increase in *Lactobacillus* might have resulted from *B. licheniformis* BCG supplementation, which creates a suitable environment for the colonization of *Lactobacillus* anaerobic bacteria by consumption of oxygen in the gut (Fazelnia et al., 2021). In previous studies, *Lactobacillus* administration has been found to contribute to nutrient absorption by significantly increasing the expression of sugar transporter genes, including *GLUT-2, GLUT-5, SGLT1*, and *SGLT4* (Faseleh Jahromi et al., 2016). *Lactobacillus* enhances occludin levels and suppresses *Escherichia coli* invasion in intestinal epithelial cells (Resta-Lenert and Barrett, 2003). Moreover, *Lactobacillus* reverses LPS-induced disruption in tight junction proteins, such as occludin, claudin-1, and ZO-1 (Zhou et al., 2014; Roselli et al., 2017). These findings were consistent with our observations indicating that *f_Lactobacillaceae* and *Lactobacillus* were significantly positively correlated with the expression of *ZO-1, claudin-1, mucin-2*, and *occludin*, and the occludin expression in the BCG2 group was significantly higher than that in the CT group. In addition, *Limosilactobacillus* spp. and *Lactobacillus* spp. have been found to significantly decrease the abundance of *Helicobacter*, one of the most common pathogens globally associated with gastritis and cancer, by 90% and 83%, respectively, in an infected mouse model (Zhao et al., 2022a). Moreover, *Limosilactobacillus* spp. administration significantly increased the prevalence of *Lactobacillus* spp. but decreased the abundance of *Desulfovibrio*. These relationships among *Limosilactobacillus, Lactobacillus, Helicobacter*, and *Desulfovibrio* were consistent with the microbial structure shift induced by *B. licheniformis* BCG administration. *Limosilactobacillus* showed a significantly positive correlation with *ZO-1* expression and α-amylase activity. These findings indicated that the increase in *f_Lactobacillaceae, Lactobacillus*, and *Limosilactobacillus* levels attributed to *B. licheniformis* BCG treatment improved intestinal barrier function through interaction with epithelial cells in broilers.

*Desulfovibrionaceae* is a family of opportunistic pathogens such as *Desulfovibrio*, which is a major sulfate-reducing bacterium that is ubiquitous in human intestines (Ichiishi et al., 2010). These sulfate-reducing bacteria destroy intestinal epithelial cells through generating large amounts of hydrogen sulfide (Zhang-Sun et al., 2015). In agreement with the current findings, *B. licheniformis* DSM5749 administration sustains intestinal health in laying hens by decreasing the *Desulfovibrio* level (Pan et al., 2022). *Alistipes* has been isolated from the appendicular, abdominal, perirectal, and brain abscesses, thus indicating a potential opportunistic pathogenic role in humans (Parker et al., 2020). A high relative abundance of *Alistipes* is strongly associated with gut disorders and host diseases, such as liver fibrosis and non-alcoholic steatohepatitis, which is inhibited by *Bacillus licheniformis* H2 administration (Parker et al., 2020; Zhao et al., 2022b). *Campylobacter* is well known as a major cause of acute bacterial enteritis in humans. Poultry is considered a major reservoir of *Campylobacter* and generally colonized by *Campylobacter* at the age of 2 weeks; therefore, *Campylobacter* may be as a principal vehicle of transmission to humans (Evans and Sayers, 2000; Lamb-Rosteski et al., 2008). Furthermore, *Vibrio*, a potential pathogen, should be harmful for host health (Richards et al., 2016). *Streptococcus* and *Escherichia coli* are pathogens that metabolize proteins in the small intestine (Ma et al., 2017). The above bacteria decreased after *B. licheniformis* BCG treatment, particularly in the BCG2 group. Dietary supplementation with *B. licheniformis* BCG significantly decreased the expression of pro-inflammatory molecules, and thus might decrease the risk of bacterial enteritis in broilers and its associated postinfectious sequelae in humans. However, in the present study, the relative abundance of *Bacteroides, Prevotellaceae*_UCG-003, and *Prevotellaceae*_NK3B31_group and *Ruminococcaceae*_UCG-005 were lower in the BCG1 and BCG2 groups than the CT group. As reported, *Prevotella* can metabolize plant cell walls and produce short chain fatty acids that benefit intestinal immunity homeostasis (Ramayo-Caldas et al., 2016). *Prevotella* and *Prevotella_9* show significantly negative correlations with levels of the pro-inflammatory cytokines IL-6 and IL-12 in the jejunum (Han et al., 2022). *Bacteroides* shows a significantly negative correlation with serum IL-6 but a positive correlation with IL-4 (Zhang et al., 2022a). The abundance of *Ruminococcaceae*, beneficial bacteria that are more commonly found in healthy people, is correlated with the production of short chain fatty acids (Koh et al., 2016; Feng et al., 2022). These reports are contrary to those from a present study reporting that bacteria whose abundance decreased after *B. licheniformis* BCG administration are positively correlated with the pro-inflammatory molecules *IL-8, TLR-4, NF-*κ*B*, or *TNF-*α expression; therefore, these findings must be verified in further study.

## 5. Conclusion

The present study indicated that dietary *B. licheniformis* BCG significantly increased jejunal and ileal α-amylase, maltase, and sucrase activity; up-regulated ileal *occludin* mRNA levels; and decreased the transcript abundance of *IL-8* and *TLR-4* in the ileum in broilers. Diets with *B. licheniformis* BCG significantly decreased bacterial community richness and diversity in the ileum but not the cecum. Dietary *B. licheniformis* BCG shaped the ileac microbiota; increased the prevalence of *f_Sphingomonadaceae, Sphingomonas*, and *Limosilactobacillus*; contributed to nutrient digestion and absorption; increased the prevalence of *f_Lactobacillaceae, Lactobacillus*, and *Limosilactobacillus*; and enhanced intestinal barrier function. In addition, dietary *B. licheniformis* BCG decreased microbial community diversity; decreased the abundance of *Desulfovibrio, Alistipes Campylobacter, Vibrio, Streptococcus*, and *Escherichia coli*-Shigella; and down-regulated expression of inflammatory molecules. Therefore, diets with *B. licheniformis* BCG contributed to broiler digestion and absorption of nutrients; enhanced the intestinal physical barrier; and decreased intestinal inflammation by decreasing microbial diversity and optimizing the microbiota structure. Our data provided a theoretical basis for *B. licheniformis* BCG application in broilers.

## Data availability statement

The datasets presented in this study can be found in online repositories. The names of the repository/repositories and accession number(s) can be found below: NCBI - PRJNA648691.

## Ethics statement

The animal study was reviewed and approved by the Institutional Animal Care and Use Committee of the Institute of Feed Research of Chinese Academy of Agricultural Sciences (FRI-CAAS20210827).

## Author contributions

YH, JW, and PY designed the experiments. XX, DL, HZ, and YH conducted experiments and collected samples. JW, XX, and HC performed sample analysis. YH analyzed the data. YH, PY, and KM wrote and revised the manuscript. All authors contributed to the article and approved the submitted version.

## Footnotes

1
http://ccb.jhu.edu/software/FLASH/

2
http://drive5.com/uparse/

3
http://www.arb-silva.de/

## References

[B1] Abdelqader A. Abuajamieh M. Hayajneh F. Al-Fataftah A. R. (2020). Probiotic bacteria maintain normal growth mechanisms of heat stressed broiler chickens. *J. Therm. Biol.* 92:102654. 10.1016/j.jtherbio.2020.102654 32888580

[B2] Aijaz S. Balda M. S. Matter K. (2006). Tight junctions: Molecular architecture and function. *Int. Rev. Cytol.* 248 261–298. 10.1016/s0074-7696(06)48005-0 16487793

[B3] Akinyemi F. T. Ding J. Zhou H. Xu K. He C. Han C. (2020). Dynamic distribution of gut microbiota during embryonic development in chicken. *Poult. Sci.* 99 5079–5090. 10.1016/j.psj.2020.06.016 32988546 PMC7598139

[B4] Antonissen G. Eeckhaut V. Van Driessche K. Onrust L. Haesebrouck F. Ducatelle R. (2016). Microbial shifts associated with necrotic enteritis. *Avian. Pathol.* 45 308–312. 10.1080/03079457.2016.1152625 26950294

[B5] Awad W. A. Hess C. Hess M. (2017). Enteric pathogens and their toxin-induced disruption of the intestinal barrier through alteration of tight junctions in chickens. *Toxins* 9:60. 10.3390/toxins9020060 28208612 PMC5331439

[B6] Broer S. (2008). Amino acid transport across mammalian intestinal and renal epithelia. *Physiol. Rev.* 88 249–286. 10.1152/physrev.00018.2006 18195088

[B7] Chen Y. C. Yu Y. H. (2020). *Bacillus* licheniformis-fermented products improve growth performance and the fecal microbiota community in broilers. *Poult. Sci.* 99 1432–1443. 10.1016/j.psj.2019.10.061 32115030 PMC7587626

[B8] Chen Z. M. Chang W. H. Zheng A. J. Zhang S. Cai H. Y. Liu G. H. (2018). Comparison of gut microbial diversity in Beijing oil and arbor acres chickens. *Rev. Bras. Cienc. Avic.* 20 37–44. 10.1590/1806-9061-2017-0549

[B9] Coe N. R. Smith A. J. Frohnert B. I. Watkins P. A. Bernlohr D. A. (1999). The fatty acid transport protein (FATP1) is a very long chain acyl-CoA synthetase. *J. Biol. Chem.* 274 36300–36304. 10.1074/jbc.274.51.36300 10593920

[B10] Collins S. M. Surette M. Bercik P. (2012). The interplay between the intestinal microbiota and the brain. *Nat. Rev. Microbiol.* 10 735–742. 10.1038/nrmicro2876 23000955

[B11] Council N. R. (1994). *Nutrient requirements of poultry: Ninth revised edition.* Washington, DC: The National Academies Press.

[B12] Deng B. Wu J. Li X. Men X. Xu Z. (2017). Probiotics and probiotic metabolic product improved intestinal function and ameliorated LPS-induced injury in rats. *Curr. Microbiol.* 74 1306–1315. 10.1007/s00284-017-1318-7 28761979

[B13] Deng W. Dong X. F. Tong J. M. Zhang Q. (2012). The probiotic *Bacillus* licheniformis ameliorates heat stress-induced impairment of egg production, gut morphology, and intestinal mucosal immunity in laying hens. *Poult. Sci.* 91 575–582. 10.3382/ps.2010-01293 22334732

[B14] Dhital S. Warren F. J. Butterworth P. J. Ellis P. R. Gidley M. J. (2017). Mechanisms of starch digestion by alpha-amylase-structural basis for kinetic properties. *Crit. Rev. Food Sci. Nutr.* 57 875–892. 10.1080/10408398.2014.922043 25751598

[B15] Duan Y. Wang Y. Dong H. Ding X. Liu Q. Li H. (2018). Changes in the intestine microbial, digestive, and immune-related genes of litopenaeus vannamei in response to dietary probiotic *Clostridium* butyricum supplementation. *Front. Microbiol.* 9:2191. 10.3389/fmicb.2018.02191 30283419 PMC6156435

[B16] Evans S. J. Sayers A. R. (2000). A longitudinal study of *Campylobacter* infection of broiler flocks in Great Britain. *Prev. Vet. Med.* 46 209–223. 10.1016/s0167-5877(00)00143-4 10913805

[B17] Faseleh Jahromi M. Wesam Altaher Y. Shokryazdan P. Ebrahimi R. Ebrahimi M. Idrus Z. (2016). Dietary supplementation of a mixture of *Lactobacillus* strains enhances performance of broiler chickens raised under heat stress conditions. *Int. J. Biometeorol.* 60 1099–1110. 10.1007/s00484-015-1103-x 26593972

[B18] Fazelnia K. Fakhraei J. Yarahmadi H. M. Amini K. (2021). Dietary supplementation of potential probiotics *Bacillus* subtilis, *Bacillus* licheniformis, and *Saccharomyces* cerevisiae and synbiotic improves growth performance and immune responses by modulation in intestinal system in broiler chicks challenged with *Salmonella* typhimurium. *Probiotics Antimicro* 13 1081–1092. 10.1007/s12602-020-09737-5 33459998

[B19] Feng S. Meng C. Hao Z. Liu H. (2022). *Bacillus* licheniformis reshapes the gut microbiota to alleviate the subhealth. *Nutrients* 14:1642. 10.3390/nu14081642 35458204 PMC9025434

[B20] Gadde U. Oh S. T. Lee Y. S. Davis E. Zimmerman N. Rehberger T. (2017). The effects of direct-fed microbial supplementation, as an alternative to antibiotics, on growth performance, intestinal immune status, and epithelial barrier gene expression in broiler chickens. *Probiotics Antimicro* 9 397–405. 10.1007/s12602-017-9275-9 28421423

[B21] Han Y. Tang C. Zhao Q. Fan S. Yang P. Zhang J. (2022). Butyrate mitigates lipopolysaccharide-induced intestinal morphological changes in weanling piglets by regulating the microbiota and energy metabolism, and alleviating inflammation and apoptosis. *Microorganisms* 10:2001. 10.3390/microorganisms10102001 36296277 PMC9606874

[B22] Han Y. Zhao Q. Tang C. Li Y. Zhang K. Li F. (2020). Butyrate mitigates weanling piglets from lipopolysaccharide-induced colitis by regulating microbiota and energy metabolism of the gut-liver axis. *Front. Microbiol.* 11:588666. 10.3389/fmicb.2020.588666 33363521 PMC7752768

[B23] Haque M. A. Wang F. Chen Y. Hossen F. Islam M. A. Hossain M. A. (2021). *Bacillus* spp. contamination: A novel risk originated from animal feed to human food chains in south-eastern Bangladesh. *Front. Microbiol.* 12:783103. 10.3389/fmicb.2021.783103 35058902 PMC8764408

[B24] Hildebrandt M. A. Hoffmann C. Sherrill-Mix S. A. Keilbaugh S. A. Hamady M. Chen Y. Y. (2009). High-fat diet determines the composition of the murine gut microbiome independently of obesity. *Gastroenterology* 137 1716–1724. 10.1053/j.gastro.2009.08.042 19706296 PMC2770164

[B25] Huang P. Zhang Y. Xiao K. Jiang F. Wang H. Tang D. (2018). The chicken gut metagenome and the modulatory effects of plant-derived benzylisoquinoline alkaloids. *Microbiome* 6:211. 10.1186/s40168-018-0590-5 30482240 PMC6260706

[B26] Huebener P. Schwabe R. F. (2013). Regulation of wound healing and organ fibrosis by toll-like receptors. *Biochim. Biophys. Acta.* 1832 1005–1017. 10.1016/j.bbadis.2012.11.017 23220258 PMC3848326

[B27] Hung D. Cheng Y. Chen W. Hua K. Pietruszka A. Dybus A. (2019). *Bacillus* licheniformis-fermented products reduce diarrhea incidence and alter the fecal microbiota community in weaning piglets. *Animals* 9:1145. 10.3390/ani9121145 31847281 PMC6940967

[B28] Ichiishi S. Tanaka K. Nakao K. Izumi K. Mikamo H. Watanabe K. (2010). First isolation of *Desulfovibrio* from the human vaginal flora. *Anaerobe* 16 229–233. 10.1016/j.anaerobe.2010.02.002 20159048

[B29] Jazi V. Mohebodini H. Ashayerizadeh A. Shabani A. Barekatain R. (2019). Fermented soybean meal ameliorates *Salmonella* typhimurium infection in young broiler chickens. *Poult. Sci.* 98 5648–5660. 10.3382/ps/pez338 31247644

[B30] Jiang Z. Zhou Y. Lu F. Han Z. Wang T. (2008). Effects of different levels of supplementary alpha-amylase on digestive enzyme activities and pancreatic amylase mRNA expression of young broilers. *Asian-Austral J. Anim. Sci.* 21 97–102. 10.5713/ajas.2008.70110

[B31] Jumpertz R. Le D. S. Turnbaugh P. J. Trinidad C. Bogardus C. Gordon J. I. (2011). Energy-balance studies reveal associations between gut microbes, caloric load, and nutrient absorption in humans. *Am. J. Clin. Nutr.* 94 58–65. 10.3945/ajcn.110.010132 21543530 PMC3127503

[B32] Kaczmarek S. A. Rogiewicz A. Mogielnicka M. Rutkowski A. Jones R. O. Slominski B. A. (2014). The effect of protease, amylase, and nonstarch polysaccharide-degrading enzyme supplementation on nutrient utilization and growth performance of broiler chickens fed corn-soybean meal-based diets. *Poult. Sci.* 93 1745–1753. 10.3382/ps.2013-03739 24864284

[B33] Kaewtapee C. Burbach K. Tomforde G. Hartinger T. Camarinha-Silva A. Heinritz S. (2017). Effect of *Bacillus* subtilis and *Bacillus* licheniformis supplementation in diets with low- and high-protein content on ileal crude protein and amino acid digestibility and intestinal microbiota composition of growing pigs. *J. Anim. Sci. Biotechnol.* 8:37. 10.1186/s40104-017-0168-2 28469845 PMC5410705

[B34] Kan L. Guo F. Liu Y. Pham V. H. Guo Y. Wang Z. (2021). Probiotics *Bacillus* licheniformis improves intestinal health of subclinical necrotic enteritis-challenged broilers. *Front. Microbiol.* 12:623739. 10.3389/fmicb.2021.623739 34084155 PMC8168541

[B35] Kim Y. Cho J. Y. Kuk J. H. Moon J. H. Cho J. I. Kim Y. C. (2004). Identification and antimicrobial activity of phenylacetic acid produced by *Bacillus* licheniformis isolated from fermented soybean, chungkook-jang. *Curr. Microbiol.* 48 312–317. 10.1007/s00284-003-4193-3 15057459

[B36] Knap I. Lund B. Kehlet A. B. Hofacre C. Mathis G. (2010). *Bacillus* licheniformis prevents necrotic enteritis in broiler chickens. *Avian. Dis.* 54 931–935. 10.1637/9106-101509-ResNote.1 20608542

[B37] Koh A. De Vadder F. Kovatcheva-Datchary P. Backhed F. (2016). From dietary fiber to host physiology: Short-chain fatty acids as key bacterial metabolites. *Cell* 165 1332–1345. 10.1016/j.cell.2016.05.041 27259147

[B38] Konieczka P. Nowicka K. Madar M. Taciak M. Smulikowska S. (2018). Effects of pea extrusion and enzyme and probiotic supplementation on performance, microbiota activity and biofilm formation in the broiler gastrointestinal tract. *Br. Poult. Sci.* 59 654–662. 10.1080/00071668.2018.1507017 30070146

[B39] Konieczka P. Sandvang D. Kinsner M. Szkopek D. Szyrynska N. Jankowski J. (2022). *Bacillus*-based probiotics affect gut barrier integrity in different ways in chickens subjected to optimal or challenge conditions. *Vet. Microbiol.* 265:109323. 10.1016/j.vetmic.2021.109323 34974377

[B40] Krajmalnik-Brown R. Ilhan Z. E. Kang D. W. DiBaise J. K. (2012). Effects of gut microbes on nutrient absorption and energy regulation. *Nutr. Clin. Pract.* 27 201–214. 10.1177/0884533611436116 22367888 PMC3601187

[B41] Kridtayopas C. Rakangtong C. Bunchasak C. Loongyai W. (2019). Effect of prebiotic and synbiotic supplementation in diet on growth performance, small intestinal morphology, stress, and bacterial population under high stocking density condition of broiler chickens. *Poult. Sci.* 98 4595–4605. 10.3382/ps/pez152 30951594

[B42] Lamb-Rosteski J. M. Kalischuk L. D. Inglis G. D. Buret A. G. (2008). Epidermal growth factor inhibits *Campylobacter* jejuni-induced claudin-4 disruption, loss of epithelial barrier function, and *Escherichia coli* translocation. *Infect. Immun.* 76 3390–3398. 10.1128/IAI.01698-07 18490463 PMC2493239

[B43] Li Q. Li L. Chen Y. Yu C. Azevedo P. Gong J. (2022a). *Bacillus* licheniformis PF9 improves barrier function and alleviates inflammatory responses against enterotoxigenic *Escherichia coli* F4 infection in the porcine intestinal epithelial cells. *J. Anim. Sci. Biotechnol.* 13:86. 10.1186/s40104-022-00746-8 35799262 PMC9264548

[B44] Li Y. Han Y. Zhao Q. Tang C. Zhang J. Qin Y. (2022b). Fermented soy and fish protein dietary sources shape ileal and colonic microbiota, improving nutrient digestibility and host health in a piglet model. *Front. Microbiol.* 13:911500. 10.3389/fmicb.2022.911500 35814707 PMC9257162

[B45] Li R. Hou G. Jiang X. Song Z. Fan Z. Hou D. X. (2019). Different dietary protein sources in low protein diets regulate colonic microbiota and barrier function in a piglet model. *Food Funct.* 10 6417–6428. 10.1039/c9fo01154d 31517363

[B46] Li S. Yan C. Liu T. Xu C. Wen K. Liu L. (2020). Research note: Increase of bad bacteria and decrease of good bacteria in the gut of layers with vs. without hepatic steatosis. *Poult. Sci.* 99 5074–5078. 10.1016/j.psj.2020.07.007 32988545 PMC7598321

[B47] Liu X. Yan H. Lv L. Xu Q. Yin C. Zhang K. (2012). Growth performance and meat quality of broiler chickens supplemented with *Bacillus* licheniformis in drinking water. *Asian-Australas J. Anim. Sci.* 25 682–689. 10.5713/ajas.2011.11334 25049614 PMC4093119

[B48] Ma N. Tian Y. Wu Y. Ma X. (2017). Contributions of the interaction between dietary protein and gut microbiota to intestinal health. *Curr. Protein Pept. Sci.* 18 795–808. 10.2174/1389203718666170216153505 28215168

[B49] Mach N. Berri M. Estelle J. Levenez F. Lemonnier G. Denis C. (2015). Early-life establishment of the swine gut microbiome and impact on host phenotypes. *Environ. Microbiol. Rep.* 7 554–569. 10.1111/1758-2229.12285 25727666

[B50] Micah H. Claire F.-L. Rob K. Gordon Jeffrey I. (2007). The human microbiome project: Exploring the microbial part of ourselves in a changing world. *Nature* 449 804–810. 10.1038/nature06244 17943116 PMC3709439

[B51] Mingmongkolchai S. Panbangred W. (2018). *Bacillus* probiotics: An alternative to antibiotics for livestock production. *J. Appl. Microbiol.* 124 1334–1346. 10.1111/jam.13690 29316021

[B52] Musa B. B. Duan Y. Khawar H. Sun Q. Ren Z. Elsiddig Mohamed M. A. (2019). *Bacillus* subtilis B21 and *Bacillus* licheniformis B26 improve intestinal health and performance of broiler chickens with *Clostridium* perfringens-induced necrotic enteritis. *J. Anim. Physiol. Anim. Nutr.* 103 1039–1049. 10.1111/jpn.13082 31016810

[B53] Neag M. A. Catinean A. Muntean D. M. Pop M. R. Bocsan C. I. Botan E. C. (2020). Probiotic *Bacillus* spores protect against acetaminophen induced acute liver injury in rats. *Nutrients* 12:632. 10.3390/nu12030632 32120994 PMC7146158

[B54] Nylund L. Satokari R. Nikkilä J. Rajilić-Stojanović M. Kalliomäki M. Isolauri E. (2013). Microarray analysis reveals marked intestinal microbiota aberrancy in infants having eczema compared to healthy children in at-risk for atopic disease. *BMC microbiol.* 13:12. 10.1186/1471-2180-13-12 23339708 PMC3563445

[B55] Oakley B. B. Lillehoj H. S. Kogut M. H. Kim W. K. Maurer J. J. Pedroso A. (2014). The chicken gastrointestinal microbiome. *FEMS Microbiol. Lett.* 360 100–112. 10.1111/1574-6968.12608 25263745

[B56] Pan X. Cai Y. Kong L. Xiao C. Zhu Q. Song Z. (2022). Probiotic effects of *Bacillus* licheniformis DSM5749 on growth performance and intestinal microecological balance of laying hens. *Front. Nutr.* 9:868093. 10.3389/fnut.2022.868093 35571886 PMC9093703

[B57] Parker B. J. Wearsch P. A. Veloo A. C. M. Rodriguez-Palacios A. (2020). The genus *Alistipes*: Gut bacteria with emerging implications to inflammation, cancer, and mental health. *Front. Immunol.* 11:906. 10.3389/fimmu.2020.00906 32582143 PMC7296073

[B58] Poulsen A. R. Jonge N. Nielsen J. L. Hojberg O. Lauridsen C. Cutting S. M. (2018). Impact of *Bacillus* spp. spores and gentamicin on the gastrointestinal microbiota of suckling and newly weaned piglets. *PLoS One* 13:e0207382. 10.1371/journal.pone.0207382 30481191 PMC6258502

[B59] Ramayo-Caldas Y. Mach N. Lepage P. Levenez F. Denis C. Lemonnier G. (2016). Phylogenetic network analysis applied to pig gut microbiota identifies an ecosystem structure linked with growth traits. *ISME J.* 10 2973–2977. 10.1038/ismej.2016.77 27177190 PMC5148198

[B60] Resta-Lenert S. Barrett K. (2003). Live probiotics protect intestinal epithelial cells from the effects of infection with enteroinvasive *Escherichia coli* (EIEC). *Gut* 52 988–997. 10.1136/gut.52.7.988 12801956 PMC1773702

[B61] Richards G. P. Fay J. P. Uknalis J. Olanya O. M. Watson M. A. (2016). Purification and host specificity of predatory *Halobacteriovorax* isolates from seawater. *Appl. Environ. Microbiol.* 82 922–927. 10.1128/AEM.03136-15 26590288 PMC4725275

[B62] Richieri G. Ogata R. Kleinfeld A. (1999). Fatty acid interactions with native and mutant fatty acid binding proteins. *Mol. Cell Biochem.* 192 77–85. 10.1007/978-1-4615-4929-1_9 10331661

[B63] Rohith H. S. Halami P. M. (2021a). The combined effect of potential probiotic *Bacillus* licheniformis MCC 2514 and *Bifidobacterium* breve NCIM 5671 towards anti-inflammatory activity on HT-29 cell lines. *Probiotics Antimicro* 10.1007/s12602-021-09851-y [Epub ahead of print]. 34581975

[B64] Rohith H. S. Halami P. M. (2021b). *In vitro* validation studies for adhesion factor and adhesion efficiency of probiotic *Bacillus* licheniformis MCC 2514 and *Bifidobacterium* breve NCIM 5671 on HT-29 cell lines. *Arch. Microbiol.* 203 2989–2998. 10.1007/s00203-021-02257-y 33772601

[B65] Roselli M. Pieper R. Rogel-Gaillard C. de Vries H. Bailey M. Smidt H. (2017). Immunomodulating effects of probiotics for microbiota modulation, gut health and disease resistance in pigs. *Anim. Feed Sci. Tech.* 233 104–119. 10.1016/j.anifeedsci.2017.07.011

[B66] Sanders M. E. Merenstein D. J. Reid G. Gibson G. R. Rastall R. A. (2019). Probiotics and prebiotics in intestinal health and disease: From biology to the clinic. *Nat. Rev. Gastroenterol Hepatol.* 16 605–616. 10.1038/s41575-019-0199-6 31296969

[B67] Song J. Xiao K. Ke Y. L. Jiao L. F. Hu C. H. Diao Q. Y. (2014). Effect of a probiotic mixture on intestinal microflora, morphology, and barrier integrity of broilers subjected to heat stress. *Poult. Sci.* 93 581–588. 10.3382/ps.2013-03455 24604851

[B68] Strandwitz P. (2018). Neurotransmitter modulation by the gut microbiota. *Brain Res.* 1693 128–133. 10.1016/j.brainres.2018.03.015 29903615 PMC6005194

[B69] van der Wielen P. W. Keuzenkamp D. A. Lipman L. J. van Knapen F. Biesterveld S. (2002). Spatial and temporal variation of the intestinal bacterial community in commercially raised broiler chickens during growth. *Microb. Ecol.* 44 286–293. 10.1007/s00248-002-2015-y 12219265

[B70] Wang J. Chen L. Li D. Yin Y. Wang X. Li P. (2008). Intrauterine growth restriction affects the proteomes of the small intestine, liver, and skeletal muscle in newborn pigs. *J. Nutr.* 138 60–66. 10.1093/jn/138.1.60 18156405

[B71] Wang J. Han Y. Cai H. Wen Z. Xu X. Zhao Z. (2022). Effects of different levels of *Bacillus* licheniformis on growth performance, immune and intestinal morphology of broilers. *China Anim. Husb. Vet. Med.* 49 4593–4603. 10.16431/j.cnki.1671-7236.2022.12.000

[B72] Wang W. Li Z. Han Q. Guo Y. Zhang B. D’Inca R. (2016). Dietary live yeast and mannan-oligosaccharide supplementation attenuate intestinal inflammation and barrier dysfunction induced by *Escherichia coli* in broilers. *Br. J. Nutr.* 116 1878–1888. 10.1017/S0007114516004116 27989252

[B73] Wang W. Wang J. Zhang H. Wu S. Qi G. (2020). Effects of *Clostridium* butyricum on production performance and intestinal absorption function of laying hens in the late phase of production. *Anim. Feed Sci. Tech.* 264:114476. 10.1016/j.anifeedsci.2020.114476

[B74] Wang Y. Du W. Lei K. Wang B. Wang Y. Zhou Y. (2017). Effects of dietary *Bacillus* licheniformis on gut physical barrier, immunity, and reproductive hormones of laying hens. *Probiotics Antimicro.* 9 292–299. 10.1007/s12602-017-9252-3 28083809

[B75] Xu Y. Yu Y. Shen Y. Li Q. Lan J. Wu Y. (2021). Effects of *Bacillus* subtilis and *Bacillus* licheniformis on growth performance, immunity, short chain fatty acid production, antioxidant capacity, and cecal microflora in broilers. *Poult. Sci.* 100:101358. 10.1016/j.psj.2021.101358 34358955 PMC8350532

[B76] Yang G. Y. Zhu Y. H. Zhang W. Zhou D. Zhai C. C. Wang J. F. (2016). Influence of orally fed a select mixture of *Bacillus* probiotics on intestinal T-cell migration in weaned MUC4 resistant pigs following *Escherichia coli* challenge. *Vet. Res.* 47:71. 10.1186/s13567-016-0355-8 27424033 PMC4947265

[B77] Yang J. Huang K. Wang J. Wu D. Liu Z. Yu P. (2021). Combined use of *Bacillus* subtilis yb-114,246 and *Bacillus* licheniformis yb-214,245 improves body growth performance of chinese huainan partridge shank chickens by enhancing intestinal digestive profiles. *Probiotics Antimicro.* 13 327–342. 10.1007/s12602-020-09691-2 32783087

[B78] Zhang R. Shi X. Chen Y. Liu J. Wu Y. Xu Y. (2022a). Multi-omics revealed the protective effects of phamnolipids in lipopolysaccharide challenged broilers. *Front. Immunol.* 13:824664. 10.3389/fimmu.2022.824664 35251004 PMC8895253

[B79] Zhang X. Hu Y. Ansari A. R. Akhtar M. Chen Y. Cheng R. (2022b). Caecal microbiota could effectively increase chicken growth performance by regulating fat metabolism. *Microb. Biotechnol.* 15 844–861. 10.1111/1751-7915.13841 34264533 PMC8913871

[B80] Zhang-Sun W. Augusto L. A. Zhao L. Caroff M. (2015). Desulfovibrio desulfuricans isolates from the gut of a single individual: Structural and biological lipid A characterization. *FEBS Lett.* 589 165–171. 10.1016/j.febslet.2014.11.042 25479086

[B81] Zhao Y. Li Z. Zhao L. Wang J. Wang F. Zhang Q. (2022a). Two novel lactic acid bacteria, *Limosilactobacillus* fermentum MN-LF23 and *Lactobacillus* gasseri MN-LG80, inhibited *Helicobacter* pylori infection in C57BL/6 mice. *Food Funct.* 13 11061–11069. 10.1039/d2fo02034c 36197065

[B82] Zhao Y. Zeng Y. Zeng D. Wang H. Sun N. Xin J. (2022b). Dietary probiotic supplementation suppresses subclinical necrotic enteritis in broiler chickens in a microbiota-dependent manner. *Front. Immunol.* 13:855426. 10.3389/fimmu.2022.855426 35371037 PMC8972058

[B83] Zhou H. Wu Y. Sun X. Yin D. Wang Y. Mahmood T. (2021). Effects of exogenous alpha-(1,4)-amylase on the utilisation of corn starch and glucose metabolism in broiler chickens. *Animal* 15:100396. 10.1016/j.animal.2021.100396 34773866

[B84] Zhou M. Zeng D. Ni X. Tu T. Yin Z. Pan K. (2016). Effects of *Bacillus* licheniformis on the growth performance and expression of lipid metabolism-related genes in broiler chickens challenged with *Clostridium* perfringens-induced necrotic enteritis. *Lipids Health Dis.* 15:48. 10.1186/s12944-016-0219-2 26957116 PMC4782583

[B85] Zhou M. Zhu J. Yu H. Yin X. Sabour P. M. Zhao L. (2014). Investigation into *in vitro* and *in vivo* models using intestinal epithelial IPEC-J2 cells and *Caenorhabditis* elegans for selecting probiotic candidates to control porcine enterotoxigenic *Escherichia coli*. *J. Appl. Microbiol.* 117 217–226. 10.1111/jam.12505 24674595

[B86] Zuo Z. Li Q. Guo Y. Li X. Huang S. Hegemann J. H. (2020). Feed-borne *Bacillus* cereus exacerbates respiratory distress in chickens infected with chlamydia psittaci by inducing haemorrhagic pneumonia. *Avian. Pathol.* 49 251–260. 10.1080/03079457.2020.1716940 31951466

